# Combined Blockade of TIGIT and CD39 or A2AR Enhances NK-92 Cell-Mediated Cytotoxicity in AML

**DOI:** 10.3390/ijms222312919

**Published:** 2021-11-29

**Authors:** Franziska Brauneck, Elisa Seubert, Jasmin Wellbrock, Julian Schulze zur Wiesch, Yinghui Duan, Tim Magnus, Carsten Bokemeyer, Friedrich Koch-Nolte, Stephan Menzel, Walter Fiedler

**Affiliations:** 1 Department of Oncology, Hematology and Bone Marrow Transplantation with Section Pneumology, Hubertus Wald University Cancer Center, University Medical Center Hamburg-Eppendorf, 20251 Hamburg, Germany; f.brauneck@uke.de (F.B.); elisa.seubert@stud.uke.uni-hamburg.de (E.S.); j.wellbrock@uke.de (J.W.); cbokemeyer@uke.de (C.B.); 2 Mildred Scheel Cancer Career Center HaTriCS4, University Medical Center Hamburg-Eppendorf, 20251 Hamburg, Germany; s.menzel@uke.de; 3 Infectious Diseases Unit, I. Department of Medicine, University Medical Center Hamburg-Eppendorf, 20251 Hamburg, Germany; j.schulze-zur-wiesch@uke.de; 4 Department of Neurology, University Medical Center Hamburg-Eppendorf, 20251 Hamburg, Germany; yinghui.duan@stud.uke.uni-hamburg.de (Y.D.); t.magnus@uke.de (T.M.); 5 Institute of Immunology, University Medical Center Hamburg-Eppendorf, 20251 Hamburg, Germany; nolte@uke.de

**Keywords:** NK cells, AML, combined blockade of TIGIT and CD39 or A2AR, NK cell-mediated cytotoxicity

## Abstract

This study aimed to characterize different natural killer (NK) cell phenotypes on bone marrow and peripheral blood cells from acute myeloid leukemia (AML) patients and healthy donors (HDs). Our data show that CD56^dim^CD16^−^ and CD56^bright^CD16^−^ NK cells represent the predominant NK cell subpopulations in AML, while the CD56^dim^CD16^+^ NK cells are significantly reduced compared to HDs. Moreover, TIGIT^+^ and PVRIG^+^ cells cluster on the CD56^dim^CD16^+^ subset whereas CD39^+^ and CD38^+^ cells do so on CD56^bright^CD16^−^ NK cells in AML. Furthermore, functional effects of (co-)blockade of TIGIT and CD39 or A2AR on NK cell functionality were analyzed. These experiments revealed that the single blockade of the TIGIT receptor results in an increased NK-92 cell-mediated killing of AML cells in vitro. Combined targeting of CD39 or A2AR significantly augments the anti-TIGIT-mediated lysis of AML cells. Our data indicate that distinct NK cell subsets in AML exhibit different immunosuppressive patterns (via the TIGIT/PVRIG receptors and the purinergic pathway). In summary, we conclude that TIGIT, CD39, and A2AR constitute relevant inhibitory checkpoints of NK cells in AML patients. A combinatorial blockade synergistically strengthens NK-92 cell-mediated cytotoxicity. As inhibitors of TIGIT, CD39, and A2AR are clinically available, studies on their combined use could be conducted in the near future.

## 1. Introduction

Human natural killer (NK) cells belong to the key effectors of cancer immune surveillance [1]. As innate lymphoid cells, they possess an intrinsic selectivity and capacity to kill cancer cells without requiring prior sensitization, which is distinct from the effector T cells of the adaptive immune system. They establish a robust cytotoxic immune response much more rapidly than T cells and have the ability to recruit other adaptive responders [2,3]. These features make NK cells promising candidates for immunotherapeutic strategies in the treatment of cancer [3]. NK cells are primarily classified according to their phenotype, based on the surface expression of CD56 and CD16 [4]. The CD3^–^CD56^dim^CD16^+^ NK cell population has been defined as cytotoxic, mainly detected in the peripheral blood from healthy individuals [5]. The CD3^–^CD56^dim^CD16^−^ and CD3^–^CD56^bright^CD16^−^ NK cells have regulatory functions by secretion of different cytokines, such as interferon-gamma (IFN-y), upon exposure to the microenvironment [6,7]. The CD56^–^CD16^+^ NK cells are defined as an unconventional NK cell subset increased in chronic infections and cancer and with significantly decreased effector functions [8,9,10].

In contrast to the single dominant T cell receptor (TCR) on T cells, NK cells express a broad array of activating and inhibitory receptors on their cell surface regulating the functional activity in response to target cells and the microenvironment [11]. A well-characterized model of NK cell activation is the ‘missing-self’ mechanism [12]. Killer cell immunoglobulin-like receptors (KIRs) bind to human leukocyte antigen (HLA) molecules to transduce inhibitory signals, thereby inhibiting NK cell activation, whereas the reduction or loss of major histocompatibility complex (MHC) or HLA expression leads to NK cell activation in a missing-self manner [2,13]. Another recognition mechanism is the antibody-dependent cellular cytotoxicity (ADCC). The NK cell receptors FcγRIIIA/CD16a and/or FcγRIIC/CD32c bind to Fc portions of antibodies that are bound to target cells, leading to NK cell activation [2,14]. Similarly to T cells, NK cells are known to downregulate their responses upon interactions if a signal persists by upregulation of inhibitory receptors [15]. Besides KIRs, NK cells can express further multiple inhibitory receptors, such as CD94/NKG2A, hepatitis A virus cellular receptor (TIM-3), and the T cell immunoreceptor with Ig and ITIM domains (TIGIT) [1,16]. Even if their functional potential has been much less studied compared to that in T cells [3], in acute myeloid leukemia (AML), it was recently found that a higher frequency of TIGIT^+^ NK cells in the blood was associated with a poorer prognosis [17]. TIGIT and the poliovirus receptor-related immunoglobulin domain-containing protein (PVRIG, CD112R) are inhibitory receptors that compete with DNAX accessory molecule-1 (DNAM-1, CD226), an activating receptor that is downregulated on NK cells in AML [18], for their ligands poliovirus receptor (PVR, CD155) and poliovirus receptor-related 2 (PVRL2, CD112). Both ligands are highly expressed by AML cells and also associated with a poor prognosis [19]. The inhibitory receptor programmed cell death-1 (PD-1) mediates functional defects in cytotoxic NK cells in various cancers [20,21]. Lymphocyte activation gene 3 (LAG-3) is expressed on activated NK cells and has been described as a negative regulator of NK cell cytokine production during chronic stimulation [22].

In addition, NK cell function is also regulated by metabolic signaling including the purinergic pathway [23]. Increased proliferation of the tumor cells and deprivation of oxygen increases utilization of adenosine triphosphate (ATP) and activation of the cancer-associated ectoenzymes ectonucleoside triphosphate diphosphohydrolase-1 (CD39) and ecto-5′-nucleotidase (CD73), which catalyze sequential dephosphorylation of ATP to adenosine monophosphate (AMP) and extracellular adenosine [24,25], whereas cyclic ADP-ribose hydrolase (CD38) catalyzes the hydrolysis of nicotinamide adenine dinucleotide (NAD) [26]. Accumulation of extracellular adenosine interacts with adenosine receptors expressed on NK cells, mediating suppressive signals most strongly via the adenosine A2A receptor (A2AR) [27,28].

NK cell-based immunotherapies have recently been proposed for various types of cancer since impaired NK cell effector functions have been described. This impaired NK cell functionality correlates with poor prognosis [29,30,31,32]. There are additional functional advantages of employing NK cells in contrast to T cells [33]. In AML, impaired NK cell function was associated with AML progress [17,34]. One of the advantages of developing allogeneic ready-to-use chimeric antigen receptor (CAR)-NK cells compared with CAR-T cells is their greater safety [35,36] because of their independence of HLA matching [13]. Results from current preclinical and early clinical studies in relapsed and refractory AML showed promising results from numerous NK cell-based immunotherapies [37,38], which can be augmented by the blockade of checkpoint receptors.

Hypothesizing that NK cells are dysfunctional in AML [10,34], in this study, we aimed to characterize “exhaustion” patterns and functionality of NK cells derived from the peripheral blood (PB) and bone marrow (BM) of patients with AML. In contrast to the well-defined T cell exhaustion, a consensus on the characteristic features of NK cell “exhaustion” is missing [15,39]. In this study, we focused on the (co-)expression of the inhibitory receptors TIGIT, PVRIG, PD-1, and LAG-3 as well as the ectonucleotidases CD39, CD73, and CD38 and we investigated whether TIGIT blockade together with blockade of the purinergic signaling can reinvigorate NK cell-mediated killing of AML blasts. Thereby, we aimed to identify suitable targets for antibody-based immunotherapeutic approaches to overcome NK cell dysfunction.

## 2. Results

### 2.1. Bone Marrow-Derived NK Cells of AML Patients Show a Shift towards CD56^bright^CD16^−^ and CD56^dim^CD16^−^ Cells and Are Associated with a Reduced CD56^dim^CD16^+^ Population

**Figure 1:**
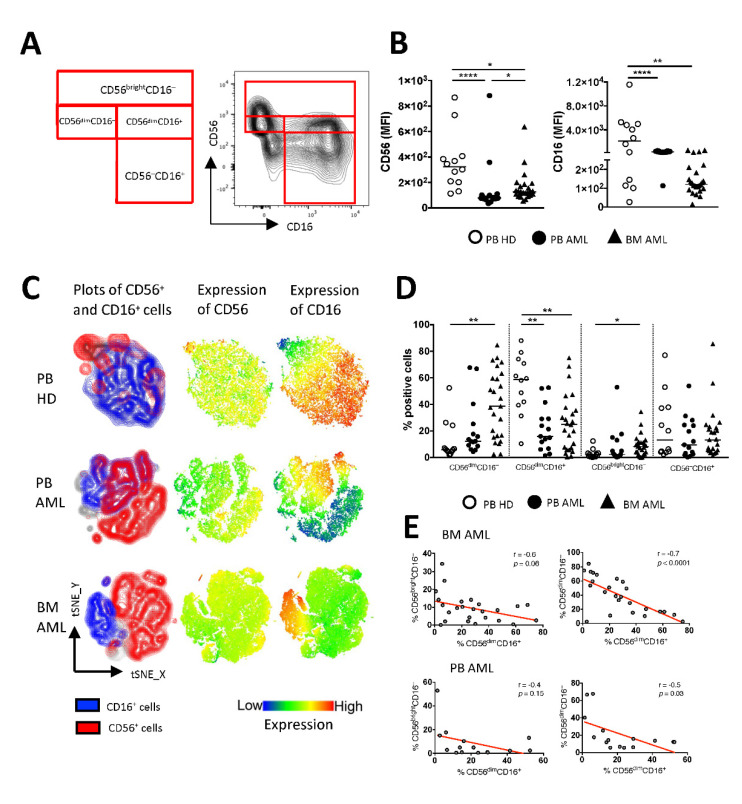
Increased frequencies of CD56^bright^CD16^−^ and CD56^dim^CD16^−^ cells are associated with a reduced CD56^dim^CD16^+^ population in AML. Flow cytometric analysis of the expression of CD56 and CD16 on mononuclear CD3^–^ cells was performed for peripheral blood (PB, *n* = 15) specimens and bone marrow (BM, *n* = 25) aspirates from patients with acute myeloid leukemia (AML) and PB-derived CD3^–^ cells from healthy donors (HDs, *n* = 12). (**A**) The gating strategy and representative flow cytometry plots show the classification of the natural killer (NK) cell subpopulations. (**B**) Summary data illustrating the median fluorescence intensity (MFI) of CD56 and CD16. (**C**) The distribution of CD56 and CD16 is depicted in t-distributed stochastic neighbor embedding (tSNE) heat maps from 4 PB samples of HDs (upper graphs), 4 PB samples of AML patients (middle graphs), and 4 BM aspirates of AML patients (lower graphs). (**D**) Summary data show the frequency of CD56^dim^CD16^−^, CD56^dim^CD16^+^, CD56^bright^CD16^−^, CD56^–^CD16^+^ NK cells from PB and BM aspirates of AML patients and PB of HDs. (**E**) Correlative analysis of the distribution of NK cell subpopulations was performed for BM- and PB-derived aspirates of the AML patients. *p* values were obtained by the ANOVA and Kruskal–Wallis test. * *p* < 0.05, ** *p* < 0.01, **** *p* < 0.0001. Pearson’s test was used to test for correlations.

Expression of the receptors CD56 and CD16 was assessed on NK cells derived from the peripheral blood (PB, *n* = 15) and bone marrow (BM, *n* = 25) aspirates from patients with untreated newly diagnosed AML and compared to PB specimens of healthy donors (HD, *n* = 12) (for the gating strategy, see Supplementary Materials Figure S1: Gating strategy). Of note, the study included only AML patients positive for at least one of the surface receptors CD117, CD34, and CD33. This selection enabled us to differentiate AML cells from NK cells. Based on their CD56 and CD16 expression, NK cells were differentiated into the following subsets (Figure 1A): the CD56^dim^CD16^−^, CD56^bright^CD16^−^, CD56^dim^CD16^+^, and CD56^–^CD16^+^ NK cells.

The phenotypic characterization of the mononuclear cells revealed that the expression of CD56 and CD16 was reduced in the PB and BM specimens from patients with AML in comparison to that in the PB of HDs (for CD56: *p* < 0.0001, *p* = 0.01 and for CD16: *p* < 0.0001, *p* = 0.0015; Figure 1B). As illustrated in the t-distributed stochastic neighbor embedding (tSNE) analyses, the distribution of NK cell subpopulations differed between patients with AML and HDs (Figure 1C). Relative to HDs, the frequency of CD56^dim^CD16^−^ and CD56^bright^CD16^−^ NK cells was increased in the BM from AML patients (*p* = 0.0027, *p* = 0.05; Figure 1D), whereas the frequency of CD56^dim^CD16^+^ NK cells was significantly decreased in the BM and the PB from AML patients (*p* = 0.0078, *p* = 0.008; Figure 1D). In addition, a low frequency of BM-derived CD56^dim^CD16^+^ NK cells correlated with higher frequencies of CD56^bright^CD16^−^ and CD56^dim^CD16^−^ NK cells (r = −0.6, *p* = 0.06 and r = −0.7, *p* < 0.0001; Figure 1E). The same tendency was seen when correlating the AML PB-derived NK cell subpopulations (Figure 1E). Taken together, in contrast to HDs, our data indicate a shift from cytotoxic to regulatory NK cells in AML.

### 2.2. NK Cells of Patients with AML Express TIGIT, PVRIG, CD39, and CD69

**Figure 2:**
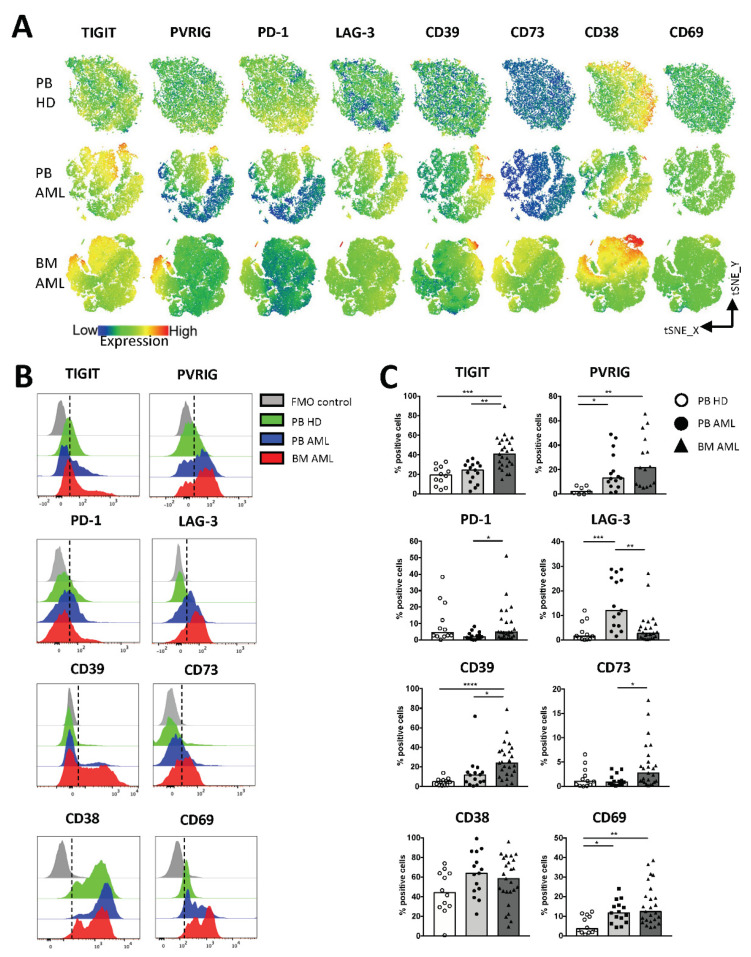
NK cells in AML highly express TIGIT, PVRIG, CD39, and CD69. The expression of TIGIT, PVRIG, PD-1, LAG-3, CD39, CD73, CD38, and CD69 was analyzed in the peripheral blood (PB, *n* = 15) and bone marrow (BM, *n* = 25, except for PVRIG *n* = 15 due to a converted multiparameter flow cytometry (MFC) panel) aspirates from patients with AML and compared with the PB (*n* = 12, for PVRIG *n* = 7) from healthy donors (HDs). (**A**) The distribution of TIGIT, PVRIG, PD-1, LAG-3, CD39, CD73, CD38, and CD69 on NK cells is depicted in tSNE heat maps analyzed for PB specimens of 4 HDs (upper graphs), 4 PB specimens of AML patients (middle graphs), and 4 BM aspirates of AML patients (lower graphs). (**B**) Representative flow cytometry plots showing the surface expression of the checkpoint molecules on NK cells from HDs (green histogram), PB-derived NK cells from AML patients (blue histogram), and BM-derived NK cells from AML patients (red histogram) versus the fluorescence minus one control (FMO, gray histogram). (**C**) Summary data illustrating the frequency of TIGIT^+^, PVRIG^+^, PD-1^+^, LAG-3^+^, CD39^+^, CD73^+^, CD38^+^, and CD69^+^ NK cells from the PB of HDs in comparison to the PB and BM aspirates of AML patients. *p* values were obtained by the ANOVA and Kruskal–Wallis test. * *p* < 0.05, ** *p* < 0.01, *** *p* < 0.001, **** *p* < 0.0001.

The (co-)expression of inhibitory receptors has been identified as characteristic feature of altered T cell functionality in cancer including in AML. Less is known about immune checkpoint molecules on NK cells as potential targets for AML immunotherapy. Thus, we assessed the surface expression of the inhibitory receptors TIGIT, PVRIG, PD-1, and LAG-3, of the ectonucleotidases CD39, CD73, and CD38 and of the activation marker CD69 on NK cells derived from the BM (*n* = 25, except for PVRIG *n* = 15) and PB (*n* = 15) from AML patients in contrast to HDs (*n* = 12, for PVRIG *n* = 7). As depicted in the tSNE plots and summary analyses (Figure 2A–C), the frequency of TIGIT^+^, PVRIG^+^, CD39^+^, and CD69^+^ NK cells was significantly increased in the BM from patients with AML in comparison to PB mononuclear cells derived from HDs (*p* = 0.0002, *p* = 0.0024, *p* < 0.0001, *p* = 0.0022). In contrast, in the PB from AML patients, TIGIT was not increased (Figure 2C). Our studies of the PB mononuclear cells show an enrichment of PVRIG^+^, LAG-3^+^, and CD69^+^ NK cells in AML in comparison to the HDs (*p* = 0.02, *p* = 0.0006, *p* = 0.0036; Figure 2C). As previously reported in the literature for some cancer types [40,41], the co-inhibitory receptor PD-1 and the ectonucleotidase CD73 were expressed by NK cells but at low levels without a significant difference between the AML and HD cohorts (Figure 2C). Analyses of the purinergic ectoenzyme CD38 revealed high numbers of positive NK cells in all three cohorts but without a significant difference (Figure 2C). Furthermore, our comparison of paired PB and BM specimens from AML patients revealed an increased frequency of TIGIT^+^, PVRIG^+^, and CD39^+^ NK cells in the BM of AML patients compared to the PB (Supplementary Materials Figure S2: Checkpoint expression on NK cells in corresponding PB- and BM-derived aspirates of AML patients). In summary, our data show an aberrant NK cell population in the PB and BM from patients with AML in comparison to HDs characterized by increased frequencies of TIGIT^+^, PVRIG^+^, CD39^+^, and CD69^+^ NK cells.

### 2.3. TIGIT, PVRIG, CD39, and CD38 Expression Is Related to the CD56^bright^CD16^−^ and the CD56^dim^CD16^+^ NK Cell Population in AML

**Figure 3:**
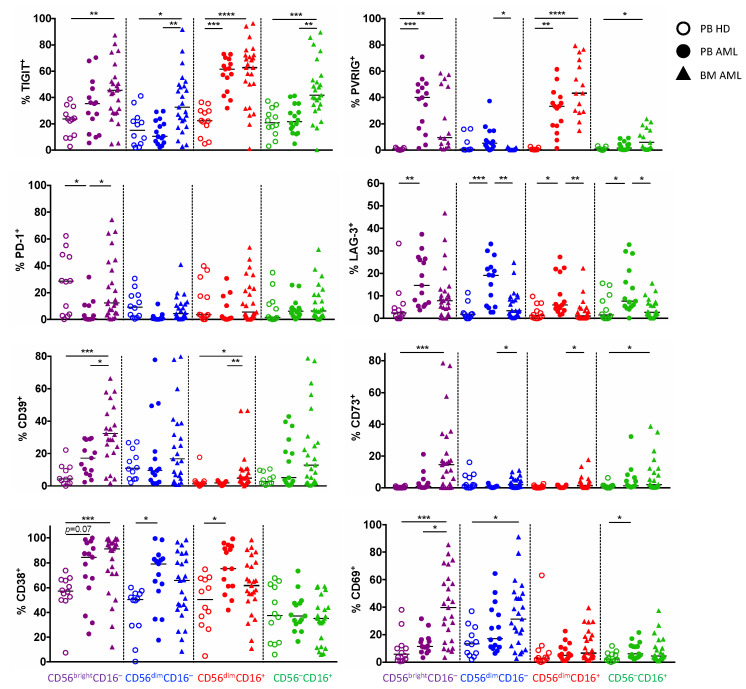
Expression of TIGIT, PVRIG, CD39, and CD38 is related to distinct NK cell populations. The expression of TIGIT, PVRIG, PD-1, LAG-3, CD39, CD73, CD38, and CD69 was compared between the different NK cell subpopulations (CD56^bright^CD16^−^, CD56^dim^CD16^−^, CD56^dim^CD16^+^, and CD56^–^CD16^+^) in the peripheral blood (PB, *n* = 15) and bone marrow (BM, *n* = 25, except for PVRIG *n* = 15 due to a converted MFC panel) from patients with AML and the PB from healthy donors (HDs, *n* = 12, for PVRIG *n* = 7). Summary data of the expression analyses on NK cell subpopulations are depicted comparing HDs vs. patients with AML. *p* values were obtained by ANOVA and Kruskal–Wallis test. * *p* < 0.05, ** *p* < 0.01, *** *p* < 0.001, **** *p* < 0.0001.

Since our analyses revealed that in AML, NK cells show a characteristic shift towards the CD56^bright^CD16^−^ and CD56^dim^CD16^−^ cells, we compared the four NK cell subpopulations regarding their expression of the inhibitory receptors, the ectonucleotidases, and the activation status in the PB and the BM. The further data refer only to results with a significant difference in the molecule expression in BM and PB aspirates from AML patients in comparison to PB from HDs. TIGIT and PVRIG were increasingly expressed by the CD56^dim^CD16^+^ subpopulation in the PB and BM from patients with AML (for TIGIT: *p* = 0.0004, *p* < 0.0001; and for PVRIG: *p* = 0.0078, *p* < 0.0001; Figure 3), whereas the expression of CD39 and CD38 was significantly upregulated on the CD56^bright^CD16^−^ cells in AML-derived PB and BM in comparison to the HD-derived PB (for CD39: *p* = 0.08, *p* < 0.0001; and for CD38: *p* = 0.07, *p* = 0.0030; Figure 3). The remaining molecules showed no clustering or differences in expression within the single NK cell subsets during the comparison of AML and HDs. Together, these subgroup analyses show an increased amount of TIGIT^+^ and PVRIG^+^ cytotoxic CD56^dim^CD16^+^ NK cells, whereas the regulatory CD56^bright^CD16^−^ population expresses higher levels of CD39 and CD38 in AML in comparison to HDs.

### 2.4. PVRIG and CD39 Are Co-Expressed with TIGIT on CD56^dim^CD16^+^ and CD56^bright^CD16^−^ NK Cells in AML

**Figure 4:**
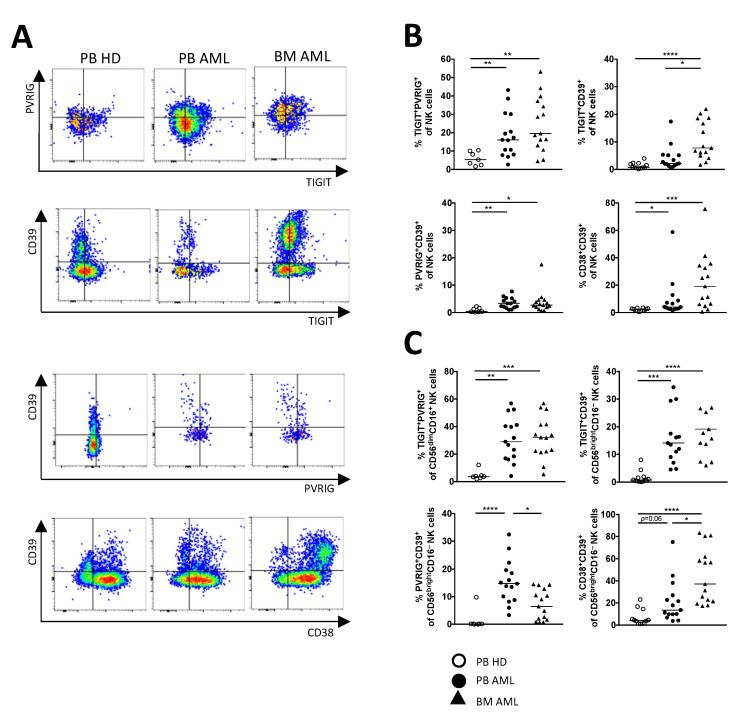
PVRIG and CD39 are co-expressed more frequently with TIGIT on NK cells in AML. Dual co-expression of the checkpoint molecules was analyzed in the peripheral blood (PB, *n* = 15) and bone marrow (BM, *n* = 25, except for PVRIG *n* = 15 due to a converted MFC panel) from AML patients and was compared with the PB from HDs (*n* = 12, for PVRIG *n* = 7). (**A**) Representative flow cytometry plots illustrate the co-expression of the checkpoint molecules. (**B**) Summary data show the co-expression of TIGIT, PVRIG, CD39, and CD38 on the total NK cell fraction. (**C**) Relevant co-expression of TIGIT, PVRIG, CD39, and CD38 is displayed on CD56^bright^CD16^−^ and CD56^dim^CD16^+^ NK cells. *p* values were obtained by the ANOVA and Kruskal–Wallis test. * *p* < 0.05, ** *p* < 0.01, *** *p* < 0.001, **** *p* < 0.0001.

Since the expression of TIGIT, PVRIG, CD39, and CD38 on NK cell subpopulations was altered in AML in comparison to HDs, we further investigated the co-expression of TIGIT, PVRIG, PD-1, LAG-3, CD39, CD73, CD38, and CD69 on the total NK cells as well as within the different NK cell subpopulations. The following data refer only to the significant results identified in both AML-derived BM and PB aspirates in comparison to HDs. As depicted in Figure 4A, our co-expression data and the analysis by tSNE (Figure 2A) discovered that the expression of CD39 and PVRIG largely overlapped with the TIGIT^+^ NK cells in AML (Figure 4B). Moreover, the highest frequencies of TIGIT^+^PVRIG^+^ cells were observed within the CD56^dim^CD16^+^ subpopulation (PB AML vs. PB HD *p* = 0.0019, BM AML vs. PB HD *p* = 0.0005; Figure 4C), whereas TIGIT^+^CD39^+^ cells were found mainly in the CD56^bright^CD16^−^ NK cell population in AML (PB AML vs. PB HD *p* = 0.0003, BM AML vs. PB HD *p* < 0.0001; Figure 4C). Additionally, we found a significant enrichment of NK cells co-expressing CD39 together with CD38 (Figure 4B). This cluster of co-expression was also located in the CD56^bright^CD16^−^ cells in AML (PB AML vs. PB HD *p* = 0.06, BM AML vs. PB HD *p* < 0.0001; Figure 4C). In conclusion, molecules of the TIGIT pathway and the purinergic signaling appear to be the most aberrant expressed checkpoint molecules on NK cells in AML. Moreover, in contrast to HDs, our analyses show that TIGIT and PVRIG are significantly co-expressed on CD56^dim^CD16^+^ cells in AML, whereas CD39 is mainly found on CD56^bright^CD16^−^ NK cells, which also co-express CD38 or TIGIT.

### 2.5. Single or Combined Checkpoint Blockade Increases NK-92 Cell-Mediated Cytotoxicity In Vitro

**Figure 5:**
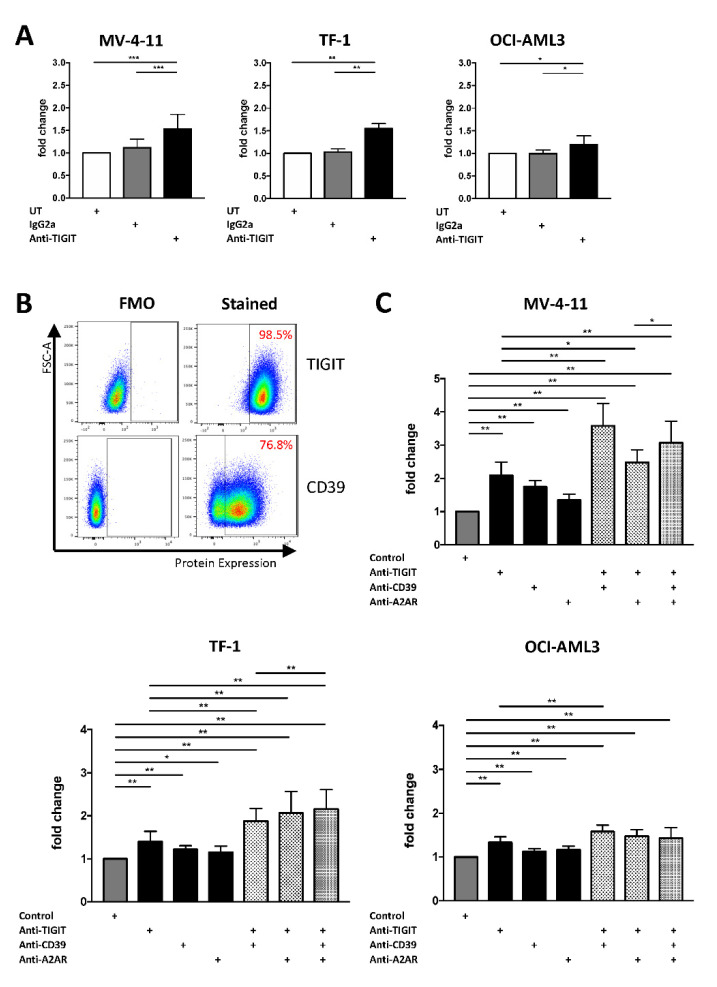
NK-92 cell-mediated cytotoxicity can be augmented by a combined blockade of TIGIT together with CD39 or A2AR. Blockade of TIGIT, CD39, and A2AR was analyzed for NK-92 cells co-cultured with AML cells over 24h in vitro. (**A**) Effects of the TIGIT blockade on NK cell-mediated lysis were analyzed by detecting the frequency of lysed AML cells that was defined by double positivity of CT green and 7-AAD measured by MFC analysis. Summary data are depicted as the mean ± SD fold changes, relative to the untreated condition for the AML cell lines MV-4-11 (*n* = 5), TF-1 (*n* = 4), OCI-AML3 (*n* = 4). (**B**) Representative flow cytometry plots showing the expression of TIGIT and CD39 on NK-92 cells. (**C**) NK cell-mediated lysis with the single blockade of TIGIT was compared with the additional administration of CD39 and A2AR blocking. Results are depicted as the mean ± SD fold changes of dead target cells, relative to the control containing the isotype controls and DMSO. Again, NK cell cytotoxicity was tested for the AML cell lines MV-4-11 (*n* = 3), TF-1 (*n* = 3), OCI-AML3 (*n* = 3). All measurements were performed in technical triplicates. *p* values were obtained by the Wilcoxon test. * *p* < 0.05, ** *p* < 0.01, *** *p* < 0.001.

In our immunophenotypic analyses, we detected that the inhibitory receptor TIGIT as well as molecules of the purinergic signaling are the most aberrant checkpoint targets on NK cells in AML. We have previously demonstrated that the ligands PVR and PVRL2 are highly expressed by AML cell lines (including MV-4-11, TF-1, and OCI-AML3) and primary AML cells [19]. Next, we analyzed the therapeutic potential of blocking TIGIT and the purinergic signaling via anti-CD39 blockade or adenosine A2A receptor (A2AR) antagonism on natural killer cells to restore the function of exhausted NK cells and improve their cytotoxic activity against AML cells. TIGIT as well as CD39 are highly expressed on the NK-92 cell line, which was used for these assays (frequency of TIGIT^+^ and CD39^+^ NK-92 cells: 98.5% and 76.8%, respectively, Figure 5B).

The single blockade of the TIGIT receptor resulted in an increased NK cell-mediated killing of AML cells after 24 h in all 3 AML cell lines (anti-TIGIT vs. IgG2a for MV-4-11 *p* = 0.001, for TF-1 *p* = 0.0078 and for OCI-AML3 *p* = 0.0156; Figure 5A). In addition, targeting of the purinergic pathway via a blockade of CD39 or A2AR augmented the NK cell-mediated lysis of the AML cells (anti-CD39 vs. control for MV-4-11 *p* = 0.0039, for TF-1 *p* = 0.0039 and for OCI-AML3 *p* = 0.0039 and anti-A2AR vs. control for MV-4-11 *p* = 0.0039, for TF-1 *p* = 0.0195 and for OCI-AML3 *p* = 0.0039; Figure 5C).

Due to these promising results, we tested the effects of the TIGIT blockade in combination with an inhibition of the purinergic pathway on NK cell-mediated killing. Dual blockade of either CD39 or A2AR together with TIGIT significantly increased AML cell lysis in 2/3 cell lines in comparison to a single blockade or control treatment (Figure 5C). Moreover, triple blockade augmented NK cell-mediated lysis of MV-4-11 cells in comparison to a dual blockade of TIGIT in combination with A2AR (Figure 5C). Triple blockade also augmented NK cell-mediated killing of the TF-1 AML cells compared to the dual inhibition of TIGIT and CD39 (Figure 5C). Further information about the significance is depicted in the Supplementary Materials Table S1: *p*-values for the differences in the fold change of 7-AAD^+^ AML cells. All effects of blockades are also analyzed and plotted as frequencies in the Supplementary Materials Figure S3: Frequencies of 7-AAD^+^ AML cells after NK cell-mediated lysis and Table S2 *p*-values for the differences in the frequency of 7-AAD^+^ AML cells.

## 3. Discussion

This study aimed to characterize NK cells in AML patients in comparison to HDs and to determine whether (co-)blockade of TIGIT and CD39 or A2AR can restore the functionality of NK cells in AML. We observed reduced cytotoxic CD56^dim^CD16^+^ NK cells in the BM and PB from AML patients compared to HDs, whereas CD56^dim^CD16^−^ and CD56^bright^CD16^−^ NK cells represent the two dominant NK cell subpopulations in AML. The presence of CD56^dim^CD16^−^ and CD56^bright^CD16^−^ NK cells was associated with an increased expression of TIGIT, PVRIG, CD39, CD38, and CD69 in AML. Interestingly, regarding the single expression of TIGIT and CD39, we observed no significant differences in the frequency of TIGIT^+^ and CD39^+^ NK cells when we compared the PB from AML patients with that of HDs, whereas the expression of LAG-3 was only increased in the PB of AML patients. However, regarding co-expression clusters unique to AML, we observed significantly increased frequencies of TIGIT^+^PVRIG^+^ NK cells located in the CD56^dim^CD16^+^ population in the PB as well as in the BM from AML patients. CD39 and CD38, on the other hand, were more frequently expressed by CD56^bright^CD16^−^ cells in AML-derived PB and BM aspirates in comparison to HDs. Due to these data, we hypothesized that distinct NK cell subsets in AML exhibited different immunosuppressive strategies (inhibitory signaling via TIGIT/PVRIG expression and immunosuppression via the adenosine-A2AR signaling). Further functional evaluation of the TIGIT axis and the purinergic signaling revealed that the single blockade of the TIGIT receptor resulted in an increased NK cell-mediated killing of AML cells after 24 h in all 3 AML cell lines. In addition, targeting of the purinergic pathway via the blockade of CD39 or A2AR augmented the NK cell-mediated lysis of AML cells. Furthermore, dual blockade of either CD39 or A2AR together with TIGIT significantly increased AML cell lysis in 2/3 cell lines in comparison to both a single blockade respectively and the control treatment. These findings implicate a synergistic effect of the combinatorial blockade of the purinergic pathway together with the TIGIT axis in AML.

In line with data in the literature, the majority of the analyzed NK cells derived from PB of HDs were further delineated as mature CD56^dim^CD16^+^ NK cells [42]. Contrary to HDs, in AML, we found a significantly altered distribution of NK cells with increased frequencies of PB- and BM-derived CD56^bright^CD16^−^ and CD56^dim^CD16^−^ NK cell populations. Overall, we observed a loss of CD56 expression on NK cells, which has also been observed by Chretien et al. [10]. In addition, a reduction of cytotoxic NK cells was recently described in different hematological malignancies (including AML) and solid tumors, especially at the tumor sites [43]. Functionally, tumor-derived NK cells were characterized by the production of the vascular endothelial growth factor (VEGF; indicating a pro-angiogenetic potential) and reduced cytotoxic effector functions [43]. Furthermore, Salomé et al. recently showed that AML-derived CD56^+^CD16^−^ NK cells produce reduced levels of granulysin, leading to an impairment in the degranulation process [44]. In light of this, we propose that aberrant NK cell populations with reduced cytotoxic functions might represent a feature of immune evasion in cancer.

NK cell function strongly depends on complex interactions between signals transmitted by activating and inhibitory receptors. Similar to T cells, NK cell-mediated anti-tumor responses were previously described to be governed by PD-1 and LAG-3 receptor signaling [21,22]. More recently, the novel inhibitory receptors TIGIT and PVRIG have also been discovered to control NK cell activation and effector function [45].

Our studies revealed that PD-1 and LAG-3 expression of NK cells was only slightly dysregulated in AML, whereas TIGIT and PVRIG were expressed on the majority of AML-derived NK cells, in particular on the cytotoxic CD56^dim^CD16^+^ NK cells. Interestingly, we observed a significant co-expression of TIGIT and PVRIG on the largest part of CD56^dim^CD16^+^ NK cells, suggesting that this NK cell subset represents the terminally activated and exhausted NK cell population in AML. Increased expression of TIGIT has previously been described for several cancer entities including AML and myelodysplastic syndrome [16,46]. Regarding the axis of CD226 and TIGIT/CD96/PVRIG, in which shared ligands and receptor-ligand affinities regulate the immune response, it was recently reported that the stimulatory receptor CD226 is downregulated on NK cells from AML patients, whereas in line with our data, an increased expression of TIGIT on T cells and NK cells was observed [19,46]. Moreover, in a cohort of 36 AML patients, the frequency of CD226^–^TIGIT^+^CD96^+^ NK cells was associated with poor prognosis of the patients [46]. Additionally, NK cell-mediated lysis of leukemias was dependent on CD226 [47]. Liu et al. also recently illustrated that TIGIT^+^ NK cells exhibit lower anti-leukemia effects, manifested by reduced cytokine production, degranulation, and cytotoxicity [17]. We confirmed these effects and extended their observations, showing that TIGIT blockade in combination with targeting the purinergic signaling could further enhance the anti-leukemic function of NK cells [17]. Our results are also in line with data from solid cancer, where blockade of TIGIT could boost functional responsiveness of NK cell subsets against ovarian cancer cell lines [48].

PVRIG, identified in 2016 [49], interacts as an inhibitory receptor with PVRL2 but not PVR. PVRL2 is highly expressed by AML cells [19] but also solid tumor cells [50], thereby adding more complexity in the TIGIT/CD226 signaling. Our data showed the expression of PVRIG^+^ NK cells (particularly within the CD56^dim^CD16^+^ subset) in the PB and BM of patients with AML. In contrast to our findings, Li et al. recently found unaltered PVRIG expression on NK cells in AML patients’ BM [51]. However, Li et al. observed significantly increased NK cell killing of PVRL2^+^ AML cell lines upon PVRIG blockade [51]. Together, these data support the relevance of TIGIT/PVRIG signaling in the suppression of anti-leukemic NK cell responses.

CD39 and CD73-generated adenosine followed by engagement of the adenosine receptor A2A additionally represents a potent immunosuppressive pathway, leading to the progression of solid tumors and hematological malignancies [28]. Concerning tumor-infiltrating NK cells, extracellular adenosine signaling via the A2AR has been shown to regulate proliferation, maturation, and cytotoxic function [52]. Here, we performed the phenotypical characterization of NK cells in AML in comparison to HDs and found an aberrant subset of CD56^bright^CD16^−^ NK cells that can be further defined by an increased frequency of cells co-expressing CD39 and CD38. Consistent with our findings, CD39 expression has been reported to be upregulated on NK cells in chronic infection (e.g., HIV infection) and solid cancer, thereby associating with a poor prognosis [53,54,55]. Moreover, Zhang et al. showed that CD39 promotes tumor progression and lung metastases by suppressing NK cells and IFN-y function in different melanoma models [55]. In addition, in CD39-deficient mice or wild-type mice treated with an inhibitor of ecto-NTPDases (POM-1), reduced formation of metastases and increased NK cell cytotoxicity were observed [55,56]. NK cell cytotoxicity could be further augmented by a combinatorial checkpoint blockade of CD39 together with A2AR or PD-1 [56].

The ectoenzyme CD38 is predominantly expressed by NK cells [57] and stimulation of CD38 with agonistic antibodies led to Ca^++^ flux, Zeta-chain-associated protein kinase 70 (ZAP70) phosphorylation, mitogen-activated protein kinase (MAPK) activation, and subsequent secretion of IFN-y [58]. Interestingly, we observed an increased frequency of CD38^+^CD56^bright^CD16^−^ NK cells in AML, which confirmed previous findings for solid cancers, where CD38-mediated adenosine production by CD56^bright^CD16^−^ NK cells accounts for a significant CD4^+^ T cell inhibition [59]. Moreover, clinical administration of the monoclonal antibody Daratumumab for myeloma patients demonstrated a depletion of CD38^high^ NK cells, leading to enhanced CD38^low/–^ NK-cell subsets with increased NK cell-dependent cytotoxicity against multiple myeloma cells [60,61].

Adenosine receptors including the A2A receptor are expressed by NK cells. Collective reports demonstrated that exogenous adenosine reduces NK cell cytotoxicity and cytokine production, particularly via the A2AR engagement. A2AR^–/–^ NK cells exert increased anti-tumor immunity and reduced tumor growth in mouse tumor models [62]. Moreover, A2AR small-molecule inhibition alone or together with a PD-L1 blockade led to tumor reduction and improved T cell and NK cell cytotoxicity [62,63].

Comparing the checkpoint expression on NK cells derived from the paired PB and BM aspirates, we observed that our data from PB were quite discrepant from the BM findings. This could be explained by a different microenvironment in the BM, which might more accurately reflect the immune milieu at the tumor site, as has been demonstrated for solid cancer studies. Williams et al. recently demonstrated similar discrepancies when comparing T cells derived from the PB with that derived from BM of AML patients [64].

Recently, increased focus has been placed on the combination of checkpoint inhibitors to achieve synergistic anti-cancer immunity [65]. Dual blockade of TIGIT and PD-1 has demonstrated enhanced anti-cancer immunity in several cancers [66,67]. Recently, it has also been described in mouse tumor models that a combined blockade of the A2AR and PD-L1 led to decreased tumor volume correlating with T cell response [63]. Though most of these studies reflected the efficacy of combinatorial blockade by exploring T cell-mediated cytotoxicity, there is growing evidence that efficacy depends on NK cells as well [68]. Up to now, in AML, the question remains as to how NK cell-mediated cytotoxicity can be improved. Here, we found that combined blockade of TIGIT together with CD39 or A2AR could significantly improve the cytotoxicity of NK cells in vitro. Because of the differential expression of the TIGIT/PVRIG axis and CD39 on different NK cell populations, the combined blockade of these pathways might augment the cytotoxic functionality of different NK cell populations in vivo. Interestingly, we observed no differences when either performing blockade of CD39 or A2AR in addition to TIGIT. This might indicate that inhibition of CD39 has no evident advantages over A2AR blockade to increase NK cell-mediated cytotoxicity. To our knowledge, ours is the first study evaluating the combinatorial effect of these two immunosuppressive pathways.

Our study has several limitations. The observations made in this study must be interpreted with caution due to the relatively small sample size of 25 patients newly diagnosed with AML and should be validated in a larger cohort. Considering the predominant expression of TIGIT and CD39 on the NK-92 cell line, further research with primary NK cells from AML patients is needed to unravel the role of TIGIT together with the purinergic signaling in AML in more detail. Moreover, the different effects of blocking CD39 or A2AR should be further investigated in more complex settings including primary cells and mouse experiments; thus, contrasting the dual impact of a CD39 blockade on increased proinflammatory ATP levels together with a reduction of extracellular adenosine with a single reduction of adenosine accumulation mediated by an A2AR inhibition.

Together, our data support the hypothesis that the inhibitory TIGIT/PVRIG receptor signaling, as well as the metabolic purinergic signaling, constitute relevant pathways for NK cell dysfunction in AML.

## 4. Materials and Methods

### 4.1. Clinical Cohorts

Peripheral blood specimens (PB, *n* = 15) and bone marrow-derived aspirates (BM, *n* = 25) were collected from patients with newly diagnosed acute myeloid leukemia (AML) before the start of intensive chemotherapeutic treatment as well as PB from age-matched healthy donors (HD, *n* = 12) after written informed consent in accordance with the Declaration of Helsinki and approval of the local ethics board of the Ärztekammer Hamburg (PV3469). All PB and BM samples were taken from patients with non-acute promyelocytic leukemia (non-APL). Paired PB and BM samples from the same patient were available for 15 of 25 AML patients. The median age of the AML patient cohort was 66 years (range 25–84), and the median age of the healthy donors was 59 years (range 41–70) (Supplementary Materials Table S3 Patient characteristics).

### 4.2. Multiparameter Flow Cytometry and Surface Staining

For multiparameter flow cytometry (MFC) analyses, cryopreserved PB and BM mononuclear cells (PBMCs, BMMCs) from patients with CD117^+^/CD34^+^/CD33^+^ AML and PBMCs of HDs were thawed in a 37 °C water bath and counted. Then, 1 × 10^6^ PBMCs or BMMCs of each specimen were washed with PBS (phosphate-buffered saline) (Dulbecco’s Phosphate Buffered Saline, Gibco Thermo Fisher Scientific, Waltham, MA, USA). After FcR blocking (FcR Blocking Reagent, human, Miltenyi Biotec, Bergisch Gladbach, Germany) for 5 min in the dark, PBMCs and BMMCs were stained with the Zombie NIR^™^ Fixable Viability Kit (BioLegend, San Diego, CA, USA) for exclusion of dead cells according to the manufacturer’s instructions. This step was followed by another washing step with PBS. For surface staining, cells were incubated with appropriate fluorochrome-conjugated antibodies including anti-CD3 (OKT3), anti-CD4 (RPA-T4), anti-CD8 (RPA-T8), anti-CD56 (5.1H11), anti-CD16 (3G8), anti-CD117 (104D2), anti-CD33 (P67.6), anti-CD34 (8G12), anti-CD19 (HIB19), anti-CD14 (63D3), anti-TIGIT (A15153G), anti-PD-1 (EH12.2H7), anti-LAG-3 (11C3C65), anti-CD39 (A1 and TU66), anti-CD73 (AD2), anti-CD38 (HIT2), anti-CD69 (FN50), anti-CD112R (W16216D), anti-CD47 (CC2C6), anti-CD138 (MI15), and anti-CD25 (M-A251) for 20 min in the dark. Antibodies were obtained from BioLegend (San Diego, CA, USA) and BD Biosciences (Franklin Lakes, NJ, USA). To wash out unbound antibodies, the cells were washed again with PBS. In the last step, the stained PBMCs and BMMCs were fixed with 0.5% paraformaldehyde (Thermo Fisher Scientific, Waltham, MA, USA) and after an incubation time of 15 min, washed again with PBS. For measuring compensation controls, single-stained BD^™^CompBeads (Anti-Mouse Ig, _k/_Negative Control Compensation Particle Set, BD Biosciences, Franklin Lakes, NJ, USA) were used. For live/dead (Zombie) compensation, compensation beads stained with anti-CD19 (APC Cy-7, BioLegend) were used. All samples were acquired on a BD FACSymphony^™^ A3 with BD FACSDiva software version 8 (BD Biosciences, Franklin Lakes, NJ, USA).

### 4.3. NK Cell-Mediated Cytotoxicity Assay

Allogeneic natural killer cell-mediated cytotoxicity assays were performed by using the NK-92 cell line as effector cells and the MV-4-11, TF-1, or OCI-AML3 cell lines as target AML cells. NK cells were co-cultured with AML cells, which were labeled with CellTracker^™^ (CT) green CMFDA (Invitrogen Thermo Fisher, Waltham, MA, USA) according to the manufacturer’s instructions in a 6:1 effector:target ratio. Co-culture was plated in a 96-well plate (1 × 10^6^ cells/mL) in NK-92 culture medium (see Section 4.4. Cell lines) and incubated with different blocking antibodies for 24 h at 37 °C and 5% CO_2_. Each well of the 96-well plate contained a 200 µL volume in total, including the blocking antibodies. For experiments with single blockade of TIGIT, co-cultured cells were incubated with 50 µg/mL anti-TIGIT antibody (Ultra-LEAF^™^ Purified anti-human TIGIT antibody clone A15153G, BioLegend, San Diego, CA, USA) or mouse IgG2a isotype control (Ultra-LEAF^™^ Purified Mouse IgG2a, _K_ Isotype Ctrl antibody, clone MG2a-53, BioLegend, San Diego, CA, USA) or left untreated. Experiments were performed in triplicates and repeated four to five times (MV-4-11 *n* = 5, TF-1 *n* = 4, OCI-AML3 *n* = 4, respectively) with the same results. Bars display the mean ± SD. For experiments with single, double, or triple blockade of TIGIT together with CD39 and of the adenosine A2A receptor, cells were co-cultured in the following conditions: control wells contained 50 µg/mL mouse IgG2a isotype control, 100 µg/mL control nanobody (the target non-binding nanobody L-10e was used in the same format as the CD39-specific nanobody [69,70,71]), and 0.01% DMSO (Sigma-Aldrich, St. Louis, MO, USA) as the A2AR antagonist was dissolved in DMSO. For the single or combinatorial checkpoint-blockade, 50µg/mL anti-TIGIT antibody (BioLegend, San Diego, CA, USA) and/or 100 µg/mL of an inhibitory anti-CD39 nanobody (the CD39-specific nanobody SB24 was generated from an immunized alpaca and reformatted into a human IgG1 heavy-chain antibody format carrying the LALAPG mutations using established protocols [69,70,71]) and/or 10 µM adenosine A2A receptor antagonist (AZD4635, kindly provided by Astra Zeneca R&D Boston, Waltham, MA, USA) were administered. In cases of a single blockade, test cultures were supplemented with the two non-corresponding controls, whereas in cases of a double blockade, test cultures were supplemented with the one non-corresponding control to make it possible to compare the results with the control wells. After 24 h of incubation, the cells of each well were collected and washed with PBS and FcR blocking was performed. After a second PBS washing step, cells were stained with 7-Aminoactinomycin D (7-AAD) Viability Staining Solution (BioLegend, San Diego, CA, USA) according to the manufacturer’s protocol. After an incubation time of 10 min at room temperature, the samples were subsequently analyzed on a BD FACSymphony^™^ A3 with BD FACSDiva software version 8 (BD Biosciences, Franklin Lakes, NJ, USA).

The frequency of AML cell lysis was determined via positivity of 7-AAD and CT green using flow cytometry. Mean values ± SD of the triplicates were calculated and plotted.

### 4.4. Cell Lines

The AML cell lines TF-1 and OCI-AML3 as well as the NK-92 were purchased from the DSMZ (Deutsche Sammlung von Mikroorganismen und Zellkulturen GmbH, Braunschweig, Germany), and the MV-4-11 cell line was purchased from ATCC (American Type Culture Collection, Manassas, VA, USA). MV-4-11 cells were cultured in RPMI 1640 (Gibco, Thermo Fisher Scientific, Waltham, MA, USA) and supplemented with 10% fetal bovine serum (FBS superior, Sigma-Aldrich, St. Louis, MO, USA). TF-1 cells were cultured in RPMI 1640 supplemented with 20% FBS and 5ng/mL human granulocyte-macrophage colony-stimulating factor (GM-CSF, PeproTech GmbH, Hamburg, Germany). OCI-AML3 cells were cultured in MEM-Alpha medium (Gibco, Thermo Fisher Scientific, Waltham, MA, USA) supplemented with 20% FBS. NK-92 cells were cultured in MEM-alpha medium supplemented with 12.5% FBS, 12.5% horse serum (Gibco, Thermo Fisher Scientific, Waltham, MA, USA), 100 U/mL Interleukin 2 (Recombinant human Interleukin-2, PeproTech GmbH, Hamburg, Germany), and 0.1 mM 2-Mercaptoethanol (Gibco, Thermo Fisher Scientific, Waltham, MA, USA). The NK-92 cells express TIGIT and CD39, which were regularly assessed by MFC. Cell cultures were incubated at 37 °C and 5% CO_2_.

### 4.5. Statistical Analysis

All flow cytometric data were analyzed using FlowJo version 10.4 and 10.5.2 software (BD Life Sciences, FlowJo, LCC, Ashland, OR, USA). Statistical analyses were carried out using Prism 8.0 (GraphPad Software, San Diego, CA, USA). All groups were tested for normal distribution with the Kolmogorov–Smirnov test. Non-normally distributed data was analyzed by the Mann–Whitney test for two unpaired groups, the Wilcoxon test for two paired groups, and the Kruskal–Wallis or Friedmann tests for more than two groups, respectively. Pearson’s correlation and Spearman’s rank correlation coefficient were applied for bivariate correlation analysis. The NK cell-mediated cytotoxicity assays were tested by the Wilcoxon test for two paired groups. Frequencies in the text are described as medians unless stated otherwise (as indicated in the figure legend). *p*-values smaller than 0.05 were considered significant, where *, **, ***, and **** indicate *p*-values between 0.01 and 0.05, 0.001 and 0.1, 0.0001 and 0.001, and <0.0001 respectively.

## 5. Conclusions

This study showed a reduced frequency of cytotoxic CD56^dim^CD16^+^ NK cells, whereas CD56^dim^CD16^−^ and CD56^bright^CD16^−^ NK cells were significantly increased in AML patients compared to HDs. Co-expression of TIGIT and PVRIG was found on the CD56^dim^CD16^+^ population and of CD39 and CD38 on CD56^bright^CD16^−^ cells in AML but not in HDs. In addition, the single blockade of the TIGIT receptor resulted in an increased NK cell-mediated killing of AML cells that can be further augmented by targeting of the purinergic pathway via a combinatorial blockade of TIGIT together with CD39 or A2AR. In summary, we conclude that TIGIT, CD39, and A2AR constitute relevant checkpoints for AML-derived NK cells. Regarding their differential expression on CD56^dim^CD16^+^ and CD56^bright^CD16^−^ NK cells, a combinatorial blockade seemed to synergistically improve anti-AML cytotoxicity. Future experiments analyzing the functional relevance of TIGIT, A2AR, and CD39 in NK cell cytotoxicity are ongoing involving primary AML cells and mouse experiments. Co-blocking TIGIT and CD39/A2AR could be a promising immunotherapeutic strategy for AML patients. Additionally, with respect to CAR-NK technologies, NK cells could be alternatively engineered to be deficient in TIGIT, CD39, or A2AR, thereby improving their antitumor effector functions. Particularly in the context that anti-TIGIT and anti-CD39/A2AR antibodies as well as small molecules are available, further (clinical) studies on their combined use need to be initiated.

## Data Availability

The datasets used and/or analyzed during the current study are available from the corresponding authors on reasonable request (f.brauneck@uke.de).

## Supplementary Materials

The following are available online at https://www.mdpi.com/article/10.3390/ijms222312919/s1.

## Abbreviations

7-AAD: 7-Aminoactinomycin D; A2AR: Adenosine A2A receptor; ADCC: Antibody-dependent cellular cytotoxicity; AML: Acute myeloid leukemia; AMP: Adenosine monophosphate; ATP: Adenosine triphosphate; BM: Bone marrow; BMMCs: Bone marrow mononuclear cells; CD38: Cyclic ADP-ribose hydrolase; CD39: Ectonucleoside triphosphate diphosphohydrolase-1; CD73: Ecto-5′-nucleotidase; CAR: Chimeric antigen receptor; CT: Cell tracker; DNAM-1: DNAX accessory molecule-1 (CD226); DNMT3A: DNA (cytosine-5)-methyltransferase 3A; ELN: European LeukemiaNet classification; FAB: French-American-British classification; FBS: Fetal bovine serum; FLT3 ITD: FMS-like tyrosine kinase 3 internal tandem duplication; FLT3 TKD: FMS-like tyrosine kinase 3 tyrosine kinase domain; FMO: Fluorescence minus one; GM-CSF: Granulocyte-macrophage colony-stimulating factor; GvHD: Graft versus host disease; HD: Healthy donor; HLA: Human leukocyte antigen; IFN-y: Interferon-gamma; KIRs: Killer-cell immunoglobulin-like receptors; LAG-3: Lymphocyte activation gene 3; MAPK: Mitogen-activated protein kinase; MFC: Multiparameter flow cytometry; MFI: Median fluorescence intensity; MHC: Major histocompatibility complex; NAD: Nicotinamide adenine dinucleotide; NK: Natural killer; non-APL: Non-acute promyelocytic leukemia; NPM1: Nucleophosmin-1; PB: Peripheral blood; PBMCs: Peripheral blood mononuclear cells; PBS: Phosphate buffered saline; PD-1: Programmed cell death-1; PD-L1: Programmed cell death-ligand 1; POM-1: Inhibitor of ecto-NTPDases; PVR: Poliovirus receptor (CD155); PVRIG: Poliovirus receptor-related immunoglobulin domain containing protein (CD112R); PVRL2: Poliovirus receptor-related 2 (CD112); TCR: T cell receptor; TIGIT: T cell immunoreceptor with Ig and ITIM domains TIM-3: Hepatitis A virus cellular receptor; TNF-a: Tumor necrosis factor alpha; tSNE: t-distributed stochastic neighbor embedding; VEGF: Vascular endothelial growth factor; ZAP70: Zeta-chain-associated protein kinase 70.
