# Supplementary material for: Combined Blockade of TIGIT and CD39 or A2AR Enhances NK-92 Cell-Mediated Cytotoxicity in AML
Source: Int J Mol Sci. 2021 Nov 29;22(23):12919. doi: 10.3390/ijms222312919 (PMC8657570; doi:10.3390/ijms222312919)
Supplement: Supplementary file 1 [file ijms-22-12919-s001.zip › ijms-1453117-supplementary.pdf]

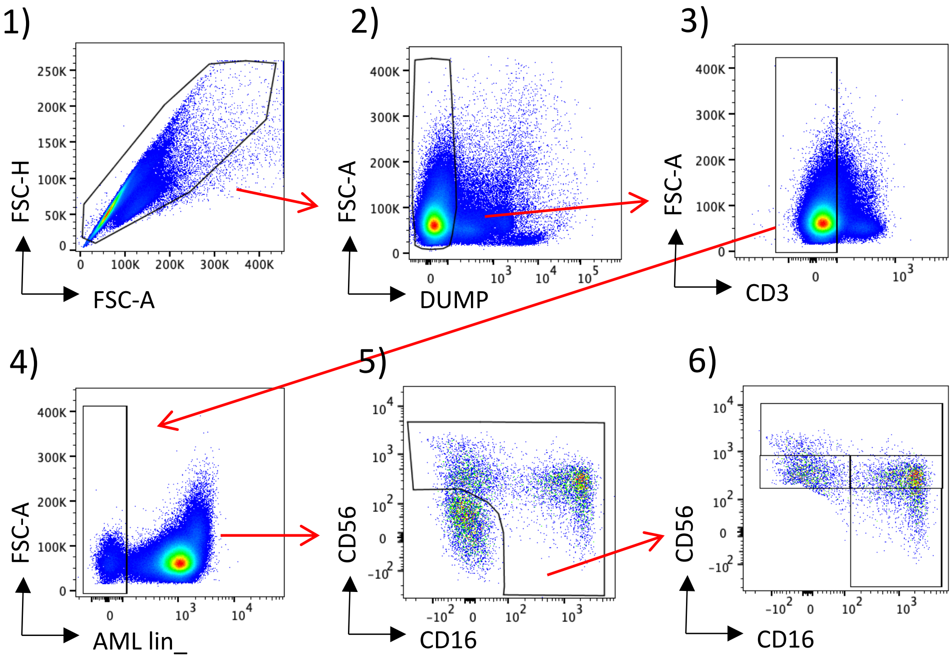

Supplementary Materials Figure S1. Gating strategy

Gating strategy used to identify $CD56^{+}CD16^{+}$ natural killer (NK) cells and their $CD56^{bright}CD16^{-}$, $CD56^{dim}CD16^{-}$, $CD56^{dim}CD16^{+}$, $CD56^{-}CD16^{+}$ subpopulations from the bone marrow (BM) and peripheral blood (PB). The same gating strategy was used to analyze samples from PB and BM: after elimination of doublets (1) and exclusion of cell debris, B cells and monocytes/macrophages via the *DUMP channel* (2), the T cells were defined as $CD3^{+}$ (3). Next, AML cells were identified on the basis of their expression of CD117, CD34, and CD33 on the $CD3^{-}$ cell subset (4). Using CD56 and CD16, NK cells were identified within the remaining cells (5). Within this population, NK cell subpopulations were defined on the basis of the co-expression pattern of CD56 and CD16 (6).

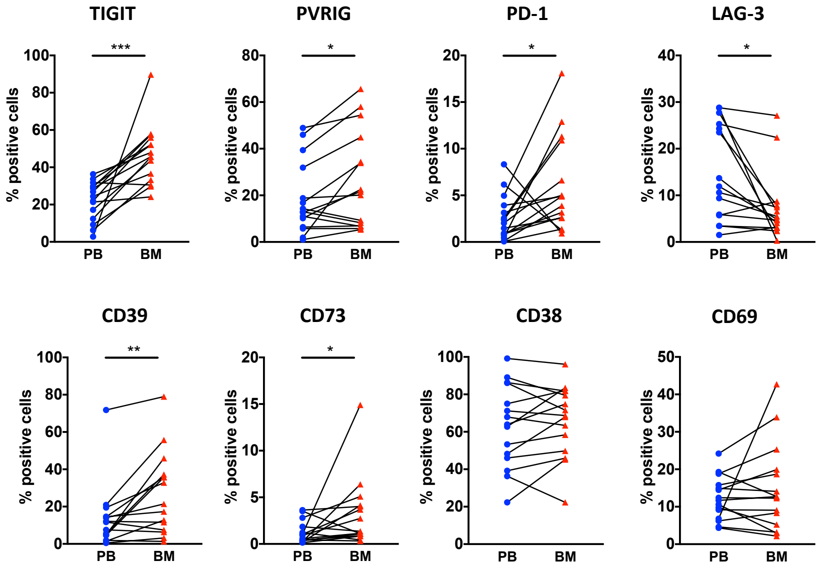

Supplementary Materials Figure S2. Checkpoint expression on NK cells in corresponding PB- and BM-derived aspirates of AML patients

The surface expression of TIGIT, PVRIG, PD-1, LAG-3, CD39, CD73, CD38, and CD69 was analyzed on NK cells derived from the peripheral blood (PB) and corresponding bone marrow (BM) from patients with newly diagnosed AML (n=15).

Summary data illustrating the frequency of positive cells on paired PB- and BM-derived NK cells in AML. P values were obtained by the Wilcoxon test. *P<0.05, **P<0.01, ***P<0.001.

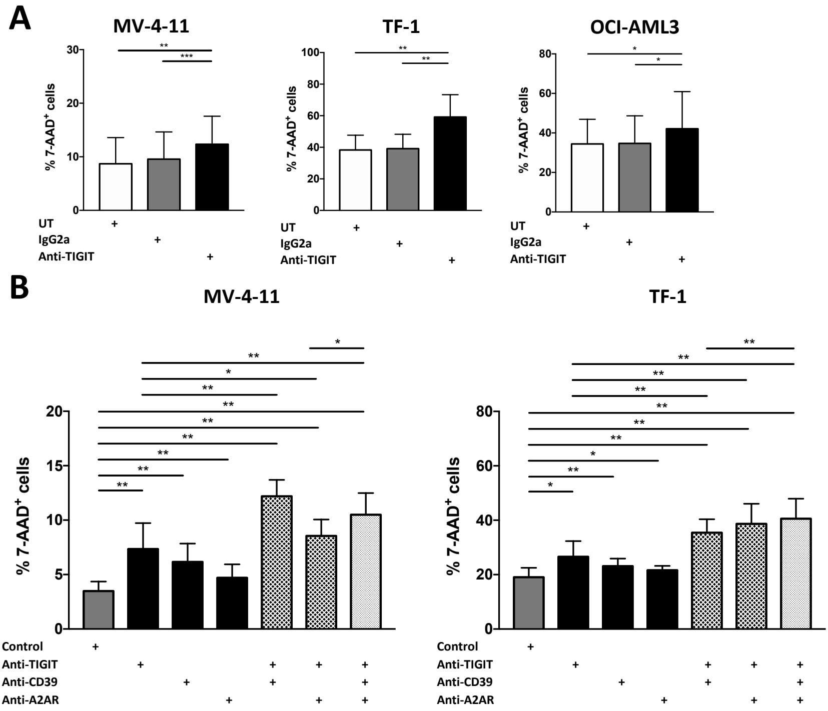

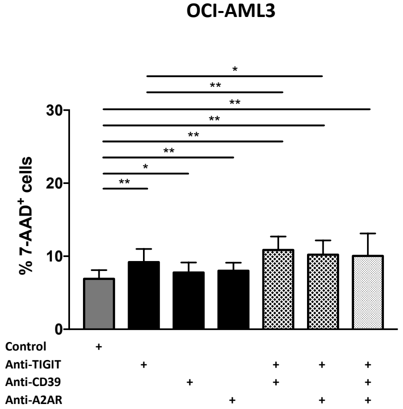

Supplementary Materials Figure S3. Frequencies of 7-AAD+ AML cells after NK cell-mediated lysis

TIGIT, CD39, and A2AR blockade was investigated for NK-92 cells co-cultured with AML cells over 24h *in vitro*. **A**) Effects of TIGIT blockade on NK cell-mediated lysis were analyzed by detecting the frequency of lysis of AML cells that was defined by double positivity of CT green and 7-AAD measured by MFC analysis. Summary data are shown as the mean ± SD frequency for the AML cell lines MV-4-11 (n=5), TF-1 (n=4), OCI-AML3 (n=4). **B**) NK cell-mediated lysis of AML cells with single blockade of TIGIT was compared with the additional administration of CD39 and A2AR blocking. Results are depicted as the mean ± SD frequencies of dead target cells, relative to the control containing the isotype controls and DMSO. MV-4-11 (n=3), TF-1 (n=3), OCI-AML3 (n=3). All measurements were performed in technical triplicates. P values were obtained by the Wilcoxon test. *P<0.05, **P<0.01, ***P<0.001.

p-value	Anti-TIGIT	Anti-CD39	Anti-A2AR	Anti-TIGIT<br>+ Anti-CD39	Anti-TIGIT<br>+ Anti-A2AR	Anti-TIGIT<br>+ Anti-CD39<br>+ Anti-A2AR
**Control**	0.0039<br>0.0078<br>0.0039	0.0039<br>0.0039<br>0.0039	0.0039<br>0.0195<br>0.0039	0.0039<br>0.0039<br>0.0039	0.0039<br>0.0039<br>0.0039	0.0039<br>0.0039<br>0.0039
**Anti-TIGIT**	-	-	-	0.0039<br>0.0039<br>0.0039	0.0195<br>0.0039<br>n.s.	0.0078<br>0.0039<br>n.s.
**Anti-TIGIT<br>+ Anti-CD39**	-	-	-	-	-	0.0039<br>0.0078<br>n.s.
**Anti-TIGIT<br>+ Anti-A2AR**	-	-	-	-	-	0.0195<br>n.s.<br>n.s.

MV-4-11
TF-1
OCI-AML3

- : not analyzed

Supplementary Materials Table S1. P-values for the differences in the fold change of 7-AAD⁺ AML cells
This table gives further information about the p-values for the significant differences in the fold change of 7-AAD⁺ AML cells (MV-4-11 (orange), TF-1 (green), and OCI-AML3 (blue)) between the various checkpoint blockades relatively to the control, depicted in **Figure 5C**. P-values smaller than 0.05 were considered significant.

p-value	Anti-TIGIT	Anti-CD39	Anti-A2AR	Anti-TIGIT<br>+ Anti-CD39	Anti-TIGIT<br>+ Anti-A2AR	Anti-TIGIT<br>+ Anti-CD39<br>+ Anti-A2AR
**Control**	0.0039<br>0.0117<br>0.0039	0.0039<br>0.0039<br>0.0117	0.0039<br>0.0391<br>0.0039	0.0039<br>0.0039<br>0.0039	0.0039<br>0.0039<br>0.0039	0.0039<br>0.0039<br>0.0039
**Anti-TIGIT**	-	-	-	0.0039<br>0.0039<br>0.0039	0.0195<br>0.0039<br>0.0391	0.0078<br>0.0039<br>n.s.
**Anti-TIGIT<br>+ Anti-CD39**	-	-	-	-	-	0.0039<br>0.0078<br>n.s.
**Anti-TIGIT<br>+ Anti-A2AR**	-	-	-	-	-	0.0117<br>n.s.<br>n.s.

MV-4-11
TF-1
OCI-AML3

- : not analyzed

Supplementary Materials Table S2. P-values for the differences in the frequency of 7-AAD⁺ AML cells
This table shows the p-values for the significant differences in the frequency (%) of 7-AAD⁺ AML cells (MV-4-11 (orange), TF-1 (green), and OCI-AML3 (blue)) between the various checkpoint blockades or the control, depicted in **Supplementary Materials Figure S3B**. P-values smaller than 0.05 were considered significant.

Patients for protein analyses	AML N_{total} = 25	HD N_{total} = 12
Age		
median	66.0 (25-84)	59.0 (41-70)
Sex		
female	13 (52%)	6 (50%)
male	12 (48%)	6 (50%)

FAB*	N_{total} = 25 (%)
M0	1 (4)
M1	10 (40)
M2	5 (20)
M3	0 (0)
M4/M4Eo	5 (20)
M5	3 (12)
M6	0 (0)
sAML: tAML	1 (4)

ELN*	N_{total} = 25 (%)
favorable	9 (36)
intermediate	8 (32)
adverse	8 (32)

Molecular genetic*	N_{total} = 25 (%)
FLT 3 ITD	9 (36)
FLT 3 TKD	4 (16)
NPM1 mut	10 (40)
DNMT3A mut**	11 (44)

*Data of 1 patient unknown
**Data of 6 patients unknown

Supplementary Materials Table S3. Patient characteristics

Age, sex, French American British classification (FAB), European LeukemiaNet classification (ELN) and the molecular aberrations: FMS like tyrosine kinase 3 internal tandem duplication or the tyrosine kinase domain mutation (FLT3 ITD or FLT 3 TKD), nucleophosmin 1 mutation (NPM1) and the DNA (cytosine-5)-methyltransferase 3A mutation (DNMT3A) of the donors who donated bone marrow (BM) aspirates or peripheral blood (PB) specimens are depicted in Table S3.
